# Right or Left: Which Is the Right Radial Access for Liver Transarterial Chemoembolization?

**DOI:** 10.3390/diagnostics15212796

**Published:** 2025-11-05

**Authors:** Francesco Giurazza, Fabio Corvino, Felice D’Antuono, Claudio Carrubba, Pietro Roccatagliata, Fortuna De Martino, Valentina Pirozzi Palmese, Tiziana Capussela, Raffaella Niola

**Affiliations:** 1 Vascular and Interventional Radiology Department, Cardarelli Hospital, Via Antonio Cardarelli 9, 80131 Naples, Italy; fabio.corvino@aocardarelli.it (F.C.); felice.dantuono@aocardarelli.it (F.D.); claudio.carrubba@aocardarelli.it (C.C.); pietro.roccatagliata@aocardarelli.it (P.R.); raffaella.niola@aocardarelli.it (R.N.); 2 Physics Unit, Department of Diagnostic-Therapeutic Advanced Technologies and Healthcare Services, Cardarelli Hospital, Via Antonio Cardarelli 9, 80131 Naples, Italy; fortuna.demartino@aocardarelli.it (F.D.M.); valentina.pirozzi@aocardarelli.it (V.P.P.); tiziana.capussela@aocardarelli.it (T.C.)

**Keywords:** radial, right, left, endovascular, access, liver, TACE, chemoembolization

## Abstract

**Objectives:** This study aims to report on radial access for transarterial chemoembolization (TACE), comparing right and left accesses in terms of technical effectiveness, safety, operator radiation exposure, and procedural comfort. **Methods:** In a single-center prospective design, patients were randomized into two groups according to right (R) or left (L) radial access. Primary endpoints were used to assess the efficacy and safety of radial access to perform liver TACE interventions; secondary endpoints were used to compare procedural comfort and operator radiation exposure. Technical efficacy was intended as procedural accomplishment via sole radial access. Safety was assessed in terms of complication occurrence. Operator radiation exposure was monitored according to dosimeters and beam-on time. Patient and operator procedural comfort was investigated using a visual analog scale. **Results:** A total of 61 patients (17 women and 44 men; mean age 68.4 years) were enrolled. Group R included 32 patients, and group L had 29; all were affected by hepatocellular carcinoma and treated with palliative or bridge-to-transplant intent. Sixteen (26.2%) had abnormal coagulation function. Technical success did not statistically differ between the two groups (96.8% group R vs. 100% group L). No major complications were recorded. While no differences were detected in terms of radiation exposure values and patient comfort, operators were significantly in favor of the right radial artery. **Conclusions:** In this sample, both right and left radial access were technically effective and safe, without significant differences in operator radiation exposure and patient comfort; considering significantly higher operator comfort with the right approach, right radial artery could be considered the *right* radial access for liver TACE interventions.

## 1. Introduction

In Interventional Radiology (IR), the right femoral artery is the most commonly adopted access for endovascular procedures; this derives from the origin, when the first procedures were performed via femoral access and catheters were developed. Since then, generations of Interventional Radiologists (IRs) have approached endovascular interventions via femoral access, developing comfortable skills with this route.

In the last decade, radial access for vascular embolization interventions has slowly gained popularity in the IR community thanks to its advantages in patient comfort and early mobility, shorter recovery time, and reduced access-related complications [1,2,3,4,5,6]. However, cardiologists have adopted radial artery access much earlier than IRs since 1989 [7], providing large comparative studies with femoral access, ending in scientific guidelines supporting the role of radial interventions for coronary procedures [8,9]. In interventional neuroradiology, radial access is also largely adopted for ambulatory diagnostic cerebral angiography, but thanks to device technology development, it is also used for intracranial procedures [10].

Dedicated radial introducer sheaths, catheters, and microcatheters have been developed by industries, facilitating the adoption of radial access by IRs, especially for abdomino-pelvic embolizations; lower profiles as well as longer lengths are currently available on the market.

Recent literature data regarding radial interventions in IR are encouraging in terms of both feasibility and safety, with the left radial artery positioned at about 90° of abduction from the body as most common set-up; the preference of the left over the right forearm comes mainly from the misconception of reducing the risk of stroke by avoiding crossing carotid trunks [6] and the shorter distance to the target. Actually, there is no significant evidence reporting an increased risk of neurological complications from right radial access [11,12]. Furthermore, right radial access positioned parallel to the trunk would present relevant advantages, specifically for abdominal visceral embolizations compared to the left arm abducted at 90°. There are easier ConeBeamCT acquisitions due to the patient standard position, quick availability of right femoral access if crossover is needed, and patient comfort without abducting the left arm or crossing over the abdomen.

Therefore, this study now aims to compare right and left radial artery access for transarterial chemoembolization (TACE) interventions, analyzing technical effectiveness, safety, operator radiation exposure, and procedural comfort.

## 2. Materials and Methods

### 2.1. Study Design

This is a single-center prospective randomized study, focusing on patients affected by hepatocellular carcinoma (HCC) and undergoing TACE via radial artery access; according to the access side, the study population was randomized into two groups (group R: right radial access; group L: left radial access).

Ethical approval from the local committee was obtained (DEL: 233/2024). Written informed consent for study participation has been acquired from all patients before the intervention.

Inclusion criteria were patients affected by HCC candidates and palliative or bridge-to-transplant TACE, availability of a recent (<30 days) thoraco-abdominal contrast-enhanced CT scan, color-doppler ultrasound (CD-US) examination of radial and ulnar arteries, and 4 weeks of follow-up.

Exclusion criteria were type 3 aortic arch, calcifications > 180° of the origin of the sovraortic trunks, Barbeau test type D, emergency conditions, upper arm dialytic fistula, and age < 18 years.

Clinical, imaging, and procedural data were retrieved from electronic datasets.

Endovascular treatments were selected according to a multidisciplinary evaluation involving hepatologists, oncologists, surgeons, nuclear physicians, and diagnostic and Interventional Radiologists.

The primary endpoints of this study were to evaluate the efficacy and safety of both radial arteries to perform TACE; the secondary endpoints were to compare procedural comfort related to the arterial access and operator radiation exposure.

### 2.2. Pre-Procedural Clinical Evaluation

All interventions were scheduled. Patients were treated in elective conditions in a in-hospital regimen with a single-night recovery in oncology, hepatology, surgery, or IR wards.

Preoperative imaging (thoraco-abdominal contrast-enhanced CT scan in all cases, with liver MR when available) and laboratory data (liver, renal, and coagulation functions with blood count) were assessed. A preoperative anesthesiological evaluation was performed.

Eventual antiplatelets and anticoagulants were suspended if clinical conditions allowed.

Platelets and fresh-frozen plasma were transfused if platelet counts and INR were <50,000/μL and >1.5, respectively, according to international guidelines [13].

### 2.3. Pre-Procedural Vascular Evaluation

The arterial anatomy was investigated at CT, assessing caliper, calcifications, origins, and angles of the subclavian, aortic arch, thoraco-abdominal aorta, and coeliac and superior mesenteric arteries.

**Figure 1:**
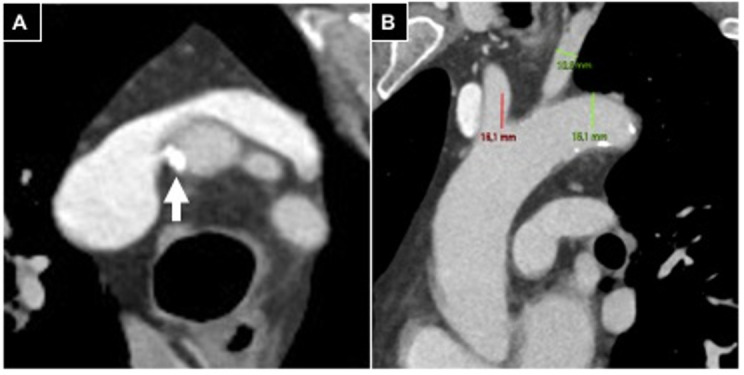
Aortic arch evaluation in a patient undergoing right radial access. (**A**) Oblique axial plane reconstructed according to the brachio-cephalic trunk, parietal calcifications involving <180° of vessel circumference (white arrow); (**B**) anterior left oblique coronal plane reconstruction, a type 2 aortic arch is detected.

In case of supraortic trunk calcifications, measurements (> or <180°) were conducted in oblique axial plane scans (Figure 1).

On the day of the intervention, radial and ulnar arteries were evaluated at CD-US using a vascular linear probe, assessing blood flow and measuring the antero-posterior vessel diameter at the level of the styloid process; if the radial artery presented a caliper <2 mm and/or the ulnar artery was occluded, radial access was excluded. The course of the radial artery from the styloid process to the elbow was evaluated as well to detect eventual vessel tortuosity and coiling.

**Figure 2:**
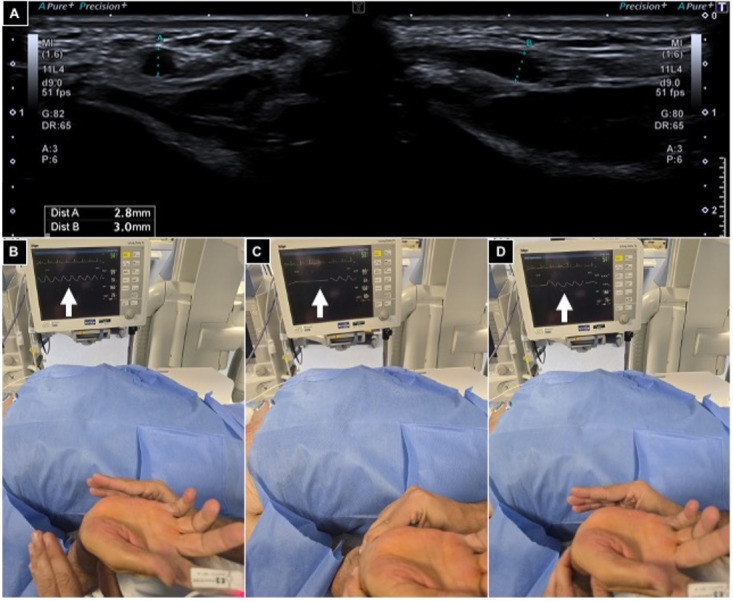
Pre-procedural radial artery evaluation. (**A**) Arterial caliper evaluation using US out of plane and in plane, showing a value > 2 mm; (**B**–**D**) Barbeau test positioning an oxygen saturimeter sensor on the right index finger and monitoring waveform variations (white arrows), free flow (**B**), compressing radial and ulnar arteries (**C**) and releasing ulnar pressure (**D**), and detecting a type A wave.

The Barbeau test was conducted by positioning a pulse oximeter on the patient’s index finger; type A-B-C findings were considered suitable for radial access (Figure 2).

### 2.4. Procedures

All interventions have been performed in an angiosuite equipped with a fixed C-arm (Axiom Artis Zee-Siemens^®^, Erlangen, Germany) by operators with >10 years of experience in liver-directed endovascular procedures and >4 years of experience in transradial interventions.

**Figure 3:**
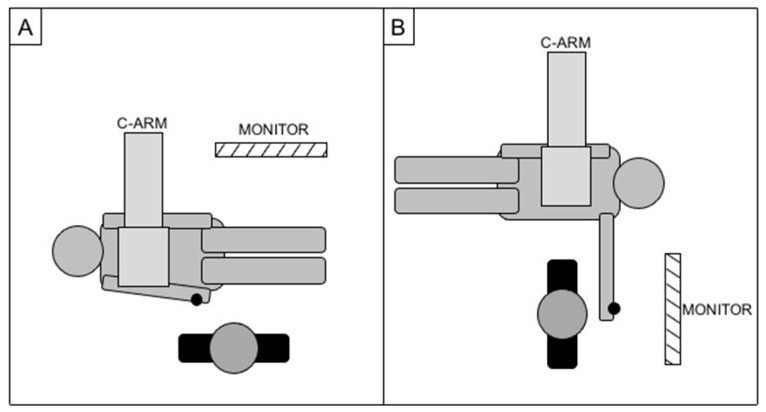
Cathlab setup for right radial access (**A**) and left radial access (**B**), with the black dot representing the site of the puncture.

In case of right access, patients were standardly positioned head first, with the right arm parallel to the trunk and the operator positioned parallel to the patient (as for a standard femoral access); in case of left access, patients were positioned feet first, with the left arm on a support at about 90° of abduction from the body while the operator was located in correspondence of the left wrist, perpendicular to the patient (Figure 3). A wrist guard was placed below the wrist to overextend the joint and superficially expose the radial artery.

Both the right radial and femoral arteries were sterilized so that, if needed, a switch to femoral access was possible.

The radial artery was punctured freehand or under US guidance, according to operator preference. Via a 25 g needle, 1 mL of local anesthesia (3% Lidocaine) was injected subcutaneously lateral to the radial artery at the level of the styloid process, massaging the site of puncture to displace local anesthesia on the radial artery surface. Using a 21 g 35 mm metallic needle, the radial artery was then punctured with an inclination of 30–45° and, after arterial blood sinking reflux, a .021″ 45 cm nitinol guidewire was advanced under fluoroscopic control to verify its straight course, avoiding collateral radial branches. Another 1 mL of local anesthesia was injected subcutaneously, and a 5Fr 10 cm introducer (Radifocus Introducer II^®^, Terumo, Japan) was inserted; 5 mL of contrast agent was injected under fluoroscopy to verify eventual spasms and confirm ulnar artery patency. To prevent vessel spasm, 2 mg of Ca-antagonist (Verapamil) diluted in 8 mL of NaCl and 500 μg of nitrates diluted in 2 mL of NaCl were intra-arterially injected via the lateral connector of the introducer. Furthermore, 2000 UI of low-molecular-weight heparin was administered endovenously if the patient had no coagulation anomalies; otherwise, heparin was avoided.

**Figure 4:**
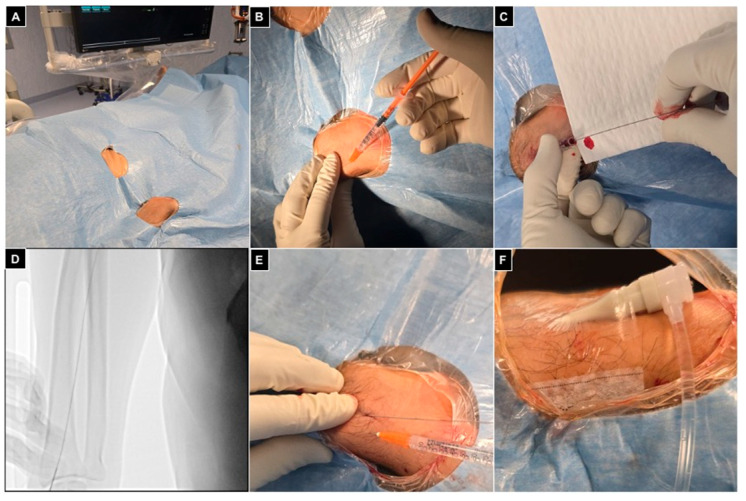
Right radial access. (**A**) Patient positioned head first, with the right arm parallel to the trunk and the wrist turned volar and hyperextended, with right femoral access sterilized and ready in case of need for crossover; (**B**) 1 mL of local anesthesia injected via a 25 g needle subcutaneously, lateral to the radial artery at the level of the styloid process; (**C**) right radial artery punctured with a 21 g needle with a .021″ 45 cm nitinol guidewire advanced inside; (**D**) fluoroscopic control showing proper guidewire positioning without collateral engagement, with compression of the access point before inserting the introducer; (**E**) a second mL of local anesthesia injected subcutaneously to reduce pain related to sheat positioning; (**F**) the 5Fr 10 cm introducer was placed and fixed to the skin with a transparent patch.

Finally, a transparent sterile patch was used to fix the introducer and avoid displacements during the procedure (Figure 4).

A 5Fr 125 cm diagnostic catheter (Multipurpose or HeadHunter tip) was advanced on a .035″ 260 cm J tip hydrophilic guidewire under roadmap guidance by looping the guidewire tip to skip radial/brachial branches; once the aortic arch was reached, a 45° left anterior oblique projection was acquired to properly depict the aortic course. If issues occurred in engaging the descending aorta, a switch to a 5Fr Pigtail tip catheter was performed, and the guidewire was advanced, opening the loop of the catheter directed into the descending aorta. The diagnostic catheter was adopted to select the coeliac trunk or the superior mesenteric artery, according to the origin of the hepatic artery. After performing DSA and ConeBeamCT (CBCT) examinations to detect lesions feeders, a 2.4Fr microcatheter (Progreat^®^, Terumo, Japan) was advanced as distal as possible to the target; chemoembolization was then performed, using the conventional (Lipiodol^®^, Guerbet, France) or degradable starch microspheres (Embocept^®^, Pharmacept, Germany) technique and Doxorubicin (50 mg diluted in 5 mL of saline) as chemotherapy.

A CBCT was acquired to depict proper lesion targeting; the .035″ guidewire was repositioned inside the diagnostic catheter before its removal to minimize the risk of clot migration into the supraortic trunks.

During CBCT acquisition, patients treated with left radial access were asked to adduce the left arm parallel to their body with consequent catheters shifting on the patient table, while with right radial access, no movements were required.

**Figure 5:**
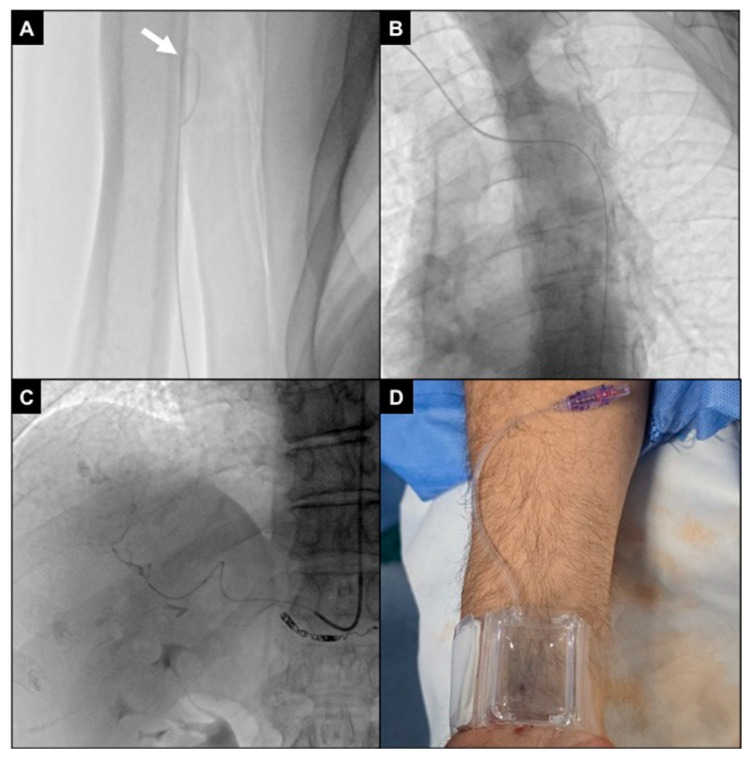
Arterial navigation. (**A**) The J tip of the .035″ hydrophilic guidewire was looped (white arrow) while navigating under fluoroscopy via the right radio-brachial axis in order to avoid collaterals; (**B**) aortic arch and descending aorta were projected in the 45° left anterior oblique plane to improve vessel route visualization; (**C**) the 5Fr multipurpose diagnostic catheter was positioned into the coeliac trunk, and the 2.4Fr microcatheter was advanced superselectively to intrahepatic segmental branches (as in this SIRT work-up for a S7 HCC lesion, after coiling of an extrahepatic gastro-epiploic feeder); (**D**) at the end of the procedure, an inflatable wristband was positioned and inflated with 20 mL of air to close the radial access.

Finally, hemostasis was obtained with a dedicated wrist band inflated with 20–22 mL of air on the site of the radial puncture; the bracelet was left for 1 h onsite and then deflated 2 mL every 10 min up to its complete removal (Figure 5).

Patients were asked to lie supine for 2 h.

### 2.5. Radiation Dose Evaluation

Radiation dose evaluation was performed according to previous studies [14,15].

Seven thermoluminescence dosimeters (TLDs) were adopted to measure the direct radiation exposure of the first operator and of the environment; overall beam-on time was also recorded.

TLDs were positioned immediately before each procedure. One TLD measured the environmental radiation dose (one TLD in the inner middle point of the C-arm); six TLDs monitored the first operator (one on the left and one on the right shoulder; one on the left and one on the right wrist; one on the left and one on the right arm of the protective glasses).

Operators were equipped with a lead apron, thyroid shield, and protective lead glasses; the lead baffle under the bed and the movable lead baffle were applied.

ALARA principles were applied to reduce radiation exposure [16].

Workload data (e.g., kV, mA) and the dose index (e.g., fluoroscopy time, kerma at interventional point) have been acquired for each procedure, using a software radiation dose index monitoring system (Physico^®^ v 1.4.22.3 by EMME ESSE M.S. SRL).

The equivalent exposure values of the first operator were obtained from the TLDs; in each procedure, data were also normalized considering and beam-on time. The operator effective dose was calculated following the Von Boetticher algorithm [17].

### 2.6. Outcome Monitoring

Technical efficacy was intended as the accomplishment of the procedural goal (chemoembolization delivery to the target confirmed at final hepatic CBCT) via sole right radial artery access, without the need to convert to femoral.

Safety was assessed in terms of complication occurrence; both minor and major access-related complications were recorded, according to the CIRSE classification standard for complications [18].

Patient comfort related to vascular access was investigated with a visual analog scale (VAS 0–10) on the day after the intervention. Patients were asked to provide an overall evaluation considering both intraprocedural vascular access pain and post-procedural comfort during the ward recovery: 0 indicated no discomfort at all, while 10 indicated maximum discomfort. Similarly, a visual analog scale (VAS 0–10) was adopted after the intervention to assess intraprocedural operator comfort in terms of catheter/microcatheter maneuvers, CBCT acquisitions, body position, and access to the femoral artery if needed.

Operator radiation exposure was monitored according to TLD values and beam-on time.

### 2.7. Follow-Up

Patients were clinically monitored for up to 4 weeks after the intervention. Radial pulse, eventual hematomas, and right-hand movements were clinically examined; if the radial pulse was not appreciable, a CD-US was conducted to confirm eventual occlusion.

### 2.8. Statistical Analysis

Continuous variables were reported as mean values and standard deviation.

Normal distribution was verified with the Kolmogorov–Smirnov test.

Regarding radiation doses derived from the TLD data comparison, the Kolmogorov–Smirnov test showed a non-normal distribution of data; therefore, a non-parametric Kruskal–Wallis test was applied with a significance threshold confidence level of 95% to assess statistical differences between variables in the two groups.

Regarding fluoroscopy time, the Kolmogorov–Smirnov test showed a normal distribution of data; therefore, a one-way ANOVA test with a significance level of 0.05 was executed to compare the two samples, while Student’s *t*-test was considered to compare mean values for each pair of groups, with a *p*-value ≤ 0.05 considered statistically significant.

All data analysis and statistical tests were performed in Excel (Microsoft^®^, USA) and R statistical software v4.4.1 environment.

## 3. Results

**Figure 6:**
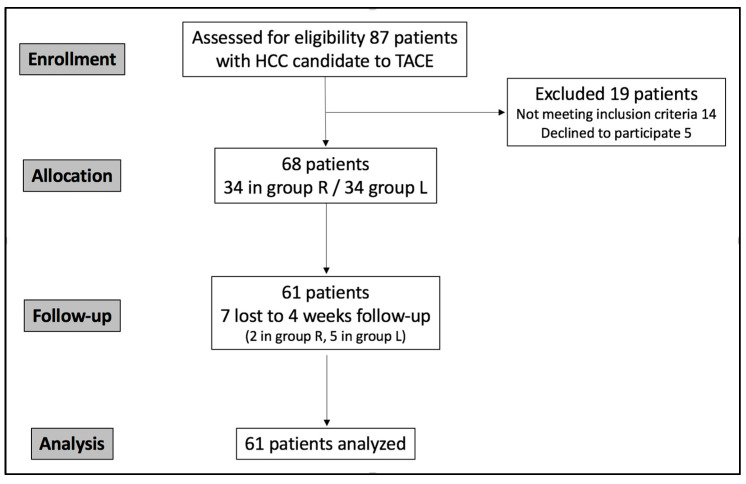
CONSORT flowchart describing study population enrollment.

The study population was recruited in a single center between September 2024 and August 2025. Eighty-seven patients were eligible; however, fourteen did not meet the inclusion criteria, and five declined to participate. Sixty-eight patients were randomized into two groups and treated with TACE; however, seven were lost to follow-up (Figure 6). The final analyzed group (Table 1) accounted for 61 consecutive patients (17 women and 44 men; mean age 68.4 years; range: 43–87): 32 in group R and 29 in group L.

Overall, 31 patients received a DSM-TACE (18 in group R and 13 in group L), and 30 received a c-TACE (14 in group R and 16 in group L).

Coagulation impairment at the time of intervention was recorded in 16 patients (overall 26.2%; 9 in group R, 28.1% and 7 in group L, 24.1%).

**Figure 7:**
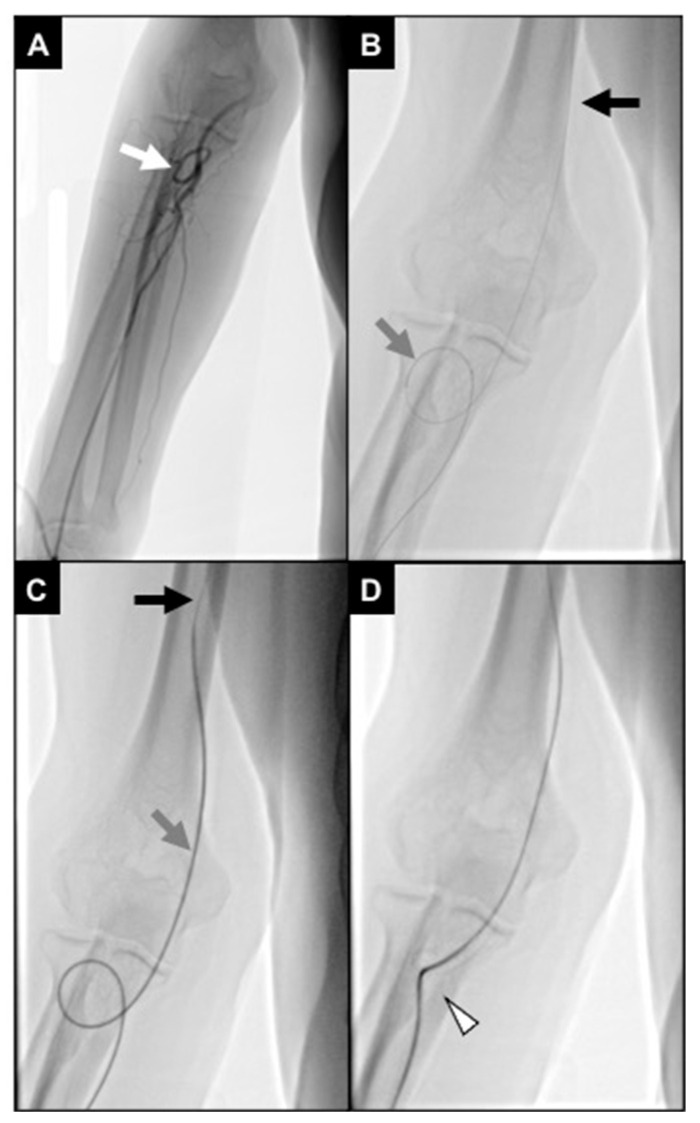
An 87-year-old man undergoing TACE for an HCC. (**A**) Initial fluoroscopic control showing distal brachial artery severe coiling (white arrow); (**B**) a 2.4Fr microcatheter (gray arrow) was navigated through the coiling, and a .018″ stiff guidewire (black arrow) was advanced up to the middle third of the brachial artery; (**C**) the microcatheter was exchanged with a 5Fr multipurpose diagnostic catheter (gray arrow), and a .035″ superstiff guidewire (black arrow) was adopted to loosen the coiling; during this maneuver, the patient referenced pain in the elbow; (**D**) brachial artery coiling was resolved; however, the vessel route remained angled (white arrowhead), not allowing proper catheter handling.

No statistical differences occurred in technical success rates (96.8% in group R and 100% in group L, *p*-value > 0.05). One patient in group R required crossover to right femoral access because of severe brachial artery coiling not allowing proper catheter handling (Figure 7).

**Figure 8:**
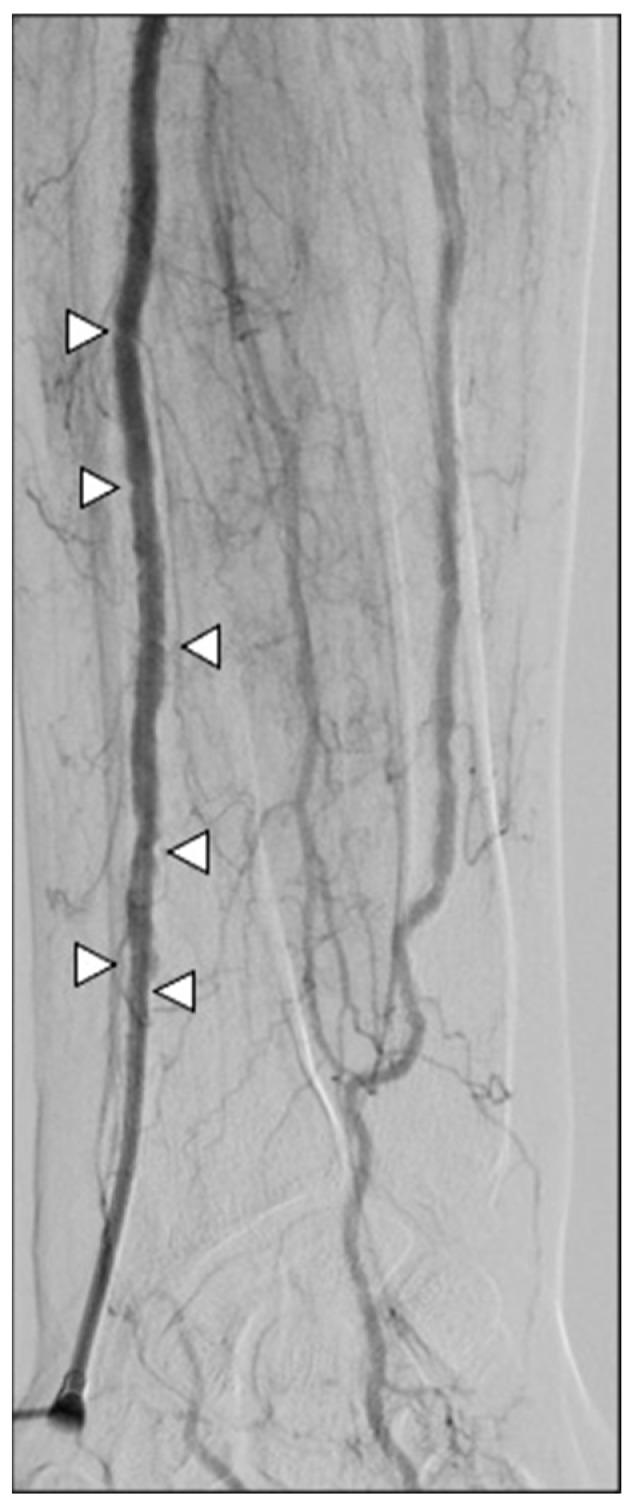
A 67-year-old man undergoing SIRT for an HCC. Severe spasm of the radial artery occurred at the end of the procedure, entraping the introducer cannula (white arrowhead); the patient referenced pain while attempting to retract the sheath, which actually did not come out. After intra-arterial injection of papaverine, removal was feasible.

In terms of safety, no major complications, such as stroke or hand ischemia, were recorded. Minor complication rates were detected in four patients (12.5%) and three patients (10.3%) in groups R and L, respectively (*p*-value > 0.05). Four patients had wrist hematoma; two patients had a painful radial spasm causing introducer entrapment and requiring intra-arterial injection of 15 mg papaverine to remove it at the end of the procedure (Figure 8); and one patient had an asymptomatic radial artery occlusion. All patients did not have clinical sequelae and did not require a prolonged hospital stay (CIRSE grade I-II).

Mean patient discomfort reported at 24 h was 1.3 (range: 0–4) in group R and 1.7 (range: 0–5) in group L (*p*-value > 0.05).

Operators expressed significantly favorable intraprocedural comfort in group R (mean values of 2.1 in group R and 5.3 in group L; *p*-value 0.03), related to CBCT acquisition and access to the femoral artery if needed.

Regarding radiation exposure, the mean fluoroscopic time was 23.0 ± 8.8 (9.1–42.7) min in group R and 23 ± 6 (14.8–33.6) min in group L; not significantly different (*p*-value > 0.05). Dosimetric data of the first operator were normalized according to fluoroscopic time and compared between the two groups (Table 2).

## 4. Discussion

In this study, right and left radial arterial accesses were compared in patients affected by HCC and treated with liver TACE interventions. Overall technical success was high, at 96.8% and 100% for procedures with right and left radial access, respectively; no major complications occurred in the whole sample, and no differences in terms of patient comfort and operator radiation exposure were recorded. On the other hand, operator procedural comfort was significantly higher with right radial access.

These findings confirm the existing literature data regarding the technical effectiveness of radial artery combined with patient comfort. In the prospective RAVI registry [1], including 99 patients, Guimaraes et al. described high safety profiles and effectiveness of left radial access for embolization interventions on fibroid, prostate, liver cancer, and hypervascular tumors without stroke, hand ischemia, and major access-related complications. Posham et al. [2] retrospectively analyzed 1500 transradial non-coronary interventions, reporting a technical success of 98.2% with a major complication rate of 0.13%. In their study, the crossover to femoral access was significantly related to female patients with a height of < 1.7 m and renal/visceral and endoleak interventions.

Multiple IR papers demonstrated increased patient comfort and preference for radial access in abdomino-pelvic embolizations [19,20,21,22,23,24] derived from the short bed rest required; especially, Yamada et al. [20] and Liu et al. [24] considered patients having received femoral and radial accesses for liver-directed procedures, reporting a strong preference for radial access.

Nevertheless, among IRs, radial access is still considered low, especially in Europe. In a survey conducted in France in 2024 [25], only 39% of enrolled centers performed radial TACE and/or SIRT; in another survey published in 2021 considering both US and European IR departments [6], 53.5% declared to adopt radial access routinely, but the largest amount of these centers was located in the US, confirming low consideration for radial access in Europe.

Furthermore, the literature data on radial IR procedures focus mainly on the left side, with right radial access being poorly investigated [15,26,27], especially for the misconception of increased stroke risk [11,12]. Different strategies have been applied in this series to reduce procedural risks, both pre-procedurally (CT examination to assess aortic arch and calcifications; radial artery evaluation in the CD-US and Barbeau test) and intraprocedurally (fluoroscopic monitoring of looped guidewire navigation via upper arm; catheter removal on guidewire to avoid clot migration). A 260 cm .035 guidewire was adopted to support eventual long catheter exchange, such as in cases where engaging the descending aorta needs to switch to a Pigtail catheter. Interestingly, Iezzi et al. [28] recently developed a pre-treatment score for safe selection of the best candidates for the transradial approach when performing liver cancer intra-arterial procedures, considering different arterial diameters and angles for left radial access.

Regarding prevention of radial spasm and occlusion, Ca-antagonists, nitrates, and eventual heparin were administered, according to the SIR guidelines for radial access [29]. Despite this, among minor complications, two spasms and one asymptomatic radial occlusion occurred, but without clinical sequelae. New pharmacological strategies are emerging to prevent radial occlusion. Aldin et al. [19] successfully administered sublingual glycerol nitrate to increase radial caliber in their series on prostate artery embolization via left radial access. Also, distal radial access in the anatomical snuffbox could be considered to minimize radial complications, but at the moment, not a lot of literature data are available, coming mainly from cardiologists [30].

Despite 26.2% of the patients having coagulation impairment, only four minor radial hematomas were recorded, confirming that radial access should be favored in patients with coagulation anomalies; indeed, the SIR guidelines [29] indicate that radial access is specifically advantageous for patients who may be at high risk of bleeding due to medical comorbidities (i.e., thrombocytopenia, chronic kidney disease, extreme body mass index, etc.) or those on therapeutic anticoagulation.

Regarding operator radiation exposure, data showed overall low exposure values, comparable with previous series considering left radial access, but with a prolonged beam-on time compared to femoral procedures [14]. No differences between right and left radial accesses were observed in this series. The literature data about radiation doses vary. Jiang H et al. [15] and Yamada et al. [20] reported statistically lower radiation exposure measured with TLDs during TACE performed via left radial access abduced 90° compared to other radial access positions and also to standard right femoral access, without differences concerning fluoroscopy time. On the other hand, Zhang et al. [21] found no differences in terms of fluoroscopy time, air kerma, and dose–area product, while Plourde et al. [31] described higher operator doses via radial access for TACE compared to femoral access. However, operator intraprocedural comfort should be considered as well, and in this study, right radial access was significantly preferred by IRs over left access. Ease of performing CBCT without moving the patient’s arm and readiness of eventual right femoral access should be considered relevant advantages over left radial access.

This study presents different limitations. First of all, its single-center design should be improved by involving operators coming from multiple IR departments; then, the number of patients treated is still too low to achieve any definitive conclusion, and larger series are certainly required. Finally, no direct comparison with femoral accesses was conducted, so no evidence of superiority can be drawn.

## 5. Conclusions

In conclusion, in this sample, both right and left radial arterial accesses for liver TACE interventions proved to be technically effective and safe, without significant differences in terms of patient comfort and operator radiation exposure; however, considering significantly higher operator intraprocedural comfort with the right approach, the right radial artery could be considered the *right* radial access for liver TACE interventions. Future multicentric studies with larger populations are needed to confirm these findings.

## Figures and Tables

**Table 1:** Population data.

Population	Right Radial (32)	Left Radial (29)
**Procedure**	14 c-TACE 18 DSM-TACE	16 c-TACE 13 DSM-TACE
**Coagulation function**	Preserved 7 antiplt/NOA tx 2 INR/PLT anomalies	Preserved 6 antiplt/NOA tx 1 INR/PLT anomalies
**Technical success**	31/32 (96.8%) (1 conversion to femoral)	29/29 (100%)
**Complications**	Major: 0 (0%) Minor: 4 (12.5%)	Major: 0 (0%) Minor: 3 (10.3%)
**Patient discomfort at 24 h** **(VAS scale 1–10)**	1.3 (range: 0–4)	1.7 (range: 0–5)
**Intraprocedural operator comfort** **(VAS scale 1–10)**	2.1 (range: 1–4)	5.3 (range: 2–7)

c-TACE: conventional transarterial chemoembolization; DSM-TACE: degradable starch microsphere transarterial chemoembolization; antiplt: antiplatelet; NOA: new oral anticoagulation; tx: therapy; INR: index-normalized ratio; PLT: platelet; VAS: visual analog scale.

**Table 2:** Radiation exposure data of the first operator according to TLDs.

	Right Radial	Left Radial	
TLD Position	*Equivalent* *Dose Normalized for FT* *[μSv/min]*	*Equivalent Dose Normalized for FT* *[μSv/min]*	*p-Value*
*TLD left shoulder*	8.1 ± 6.4 (1.4–27.9)	6.59 ± 4.9 (1.99–18.85)	0.756
*TLD right shoulder*	4.2 ± 2.5 (1.4–10.9)	4.67 ± 3.48 (1.99–14.38)	0.868
*TLD left wrist*	12.5 ± 21.2 (0.7–98.9)	13.54 ± 12.22 (0.59–49.21)	0.696
*TLD right wrist*	2 ± 2.4 (0.5–11.4)	4.35 ± 6.54 (0.63–28.9)	0.542
*Left arm glasses*	4.2 ± 4.5 (0.74–18)	3.86 ± 3.94 (0.59–15.13)	0.964
*Right arm glasses*	1.1 ± 0.5 (0.5–2.3)	3.27 ± 4.34 (0.59–18.15)	0.078

TLD: thermoluminescence dosimeter; μSv: microsievert; FT: fluoroscopic time.

## Data Availability

Research data are available upon request to the corresponding author.
